# Model Free Localization with Deep Neural Architectures by Means of an Underwater WSN

**DOI:** 10.3390/s19163530

**Published:** 2019-08-13

**Authors:** Juan Parras, Santiago Zazo, Iván A. Pérez-Álvarez, José Luis Sanz González

**Affiliations:** 1 Information Processing and Telecommunications Center, Universidad Politécnica de Madrid, ETSI Telecomunicación, Av. Complutense 30, 28040 Madrid, Spain; 2 Institute for Technological Development and Innovation in Communications (IDeTIC), Universidad de Las Palmas de Gran Canaria, 35017 Las Palmas, Spain

**Keywords:** deep learning, underwater localization, acoustic

## Abstract

In recent years, there has been a significant effort towards developing localization systems in the underwater medium, with current methods relying on anchor nodes, explicitly modeling the underwater channel or cooperation from the target. Lately, there has also been some work on using the approximation capabilities of Deep Neural Networks in order to address this problem. In this work, we study how the localization precision of using Deep Neural Networks is affected by the variability of the channel, the noise level at the receiver, the number of neurons of the neural network and the utilization of the power or the covariance of the received acoustic signals. Our study shows that using deep neural networks is a valid approach when the channel variability is low, which opens the door to further research in such localization methods for the underwater environment.

## 1. Introduction

In the last few years, there has been substantial development of wireless sensor networks (WSN). A class of WSN that has received a lot of attention lately are Acoustic Underwater WSN (AUWSN). This class of WSN is used for a variety of tasks, ranging from monitoring applications, disaster management and recovery and assisted navigation, to military related applications, as a recent survey has shown [1]. Several of these applications require a localization mechanism, which is used for different purposes, such as data tagging, node tracking, target detection or to improve the performance of communication protocols [2]. Thus, it is no surprise that a lot of attention and research have addressed the localization problem in underwater environments.

In the reviews [2,3,4,5], the authors propose several classifications for current localization methods in AUWSN. These methods rely on acoustic signals, as they have a considerably lower attenuation in the underwater channel than electromagnetic signals. The localization techniques can be based on time of arrival, angle of arrival or received power [2]. Since the underwater channel presents a time-varying path loss and a strong multipath effect, the received power measurements present a large variance, and hence are usually not used for localization [3], an exception being [6], where a hybrid method using power measurements and time of arrival is used. Still, the majority of current localization techniques rely on time of arrival [2] and usually make use of anchor nodes or active messaging.

Anchor nodes are sensors that know their localization a priori and use that information for target localization purposes. Even though anchor nodes are common [7], they introduce a deployment cost. One solution to this problem is the use of self-localization algorithms [8]. Another solution is the use of an anchor-less localization mechanism that relies on fingerprinting [9], but this method needs to model the environment. Active messaging means that the sensors, as well as the target to locate, communicate among them in order to obtain the localization, which means having an increase in battery consumption and having a cooperative target.

However, recent advances in deep learning are using the powerful modeling capabilities of Deep Neural Networks (DNNs) in order to use acoustic signal measurements in order to locate, as it is done in other communication channels [10,11]. For instance, the authors in [12,13] make use of a DNN in order to locate using the covariance matrix of the received sound pressure levels. Such approaches need a training stage but present a significant set of advantages: we may not need synchronization among sensors nor active messaging schemes, we may not need the sensor localization and there is no need to have an exhaustive a priori knowledge of the environment parameters, which means that a DNN approach could be considered model-free as it learns the mapping between the acoustic data and the localization of the source.

In this work, we give a step forward towards studying the feasibility of using model-free localization methods in underwater channels based on DNN. As we have indicated, most localization methods rely on time of arrival measurements, but recently methods based on DNN have been used to locate using the received signal. In this work, we provide the following contributions:

- We study the localization accuracy of a simple DNN using the underwater acoustic channel characterization proposed in [14], which models a time-varying shallow water acoustic channel, which takes into account several propagation effects such as multipath or frequency-dependent path loss. It is a realistic channel model, which allows us to test the accuracy of the DNN approach under different propagation conditions.
- We make use of a recurrent neural networks architecture to locate, studying the results obtained for different sizes of the neural network in order to evaluate how this influences the localization precision. As recurrent neural networks have memory, they not only learn to locate, but also to track the target, as [15] shows.
- We compare the localization accuracy using both power measurements and the covariance matrix, for several channel parameters, DNN architectures and Signal to Noise Ratio (SNR) values. To the best of our knowledge, ours is the first work in which the DNN approach is put to the test under all these conditions, which allows us to draw conclusions on both the precision and the convenience of using DNN for underwater localization. Currently, covariance measurements are used for underwater location, but we show that, under certain conditions, power measurements can be enough for underwater localization. This opens the door to new localization methods that use received signal power as input.

In short, our work provides a study on the utilization of DNNs as a localization method in underwater channels. The rest of the paper goes as follows: in Section 2, we introduce models and concepts that are used throughout the paper. Later, Section 3 thoroughly details the problem that we address. Then, in Section 4, we present our results and analyze them. Finally, we draw some conclusions in Section 5.

## 2. Background

In his section, we introduce both the channel model that we use in our work and a short introduction to feedforward neural networks.

### 2.1. Channel Model

The task of modeling the underwater acoustic channel is not easy. There are many models proposed, such as [16,17,18,19]. In each of these works, the channel random variations are adjusted differently, which gives rise to different channel models. Very remarkably, the authors from [14] propose using a statistical characterization of the underwater acoustic channels that classifies the channel variations into small and large scale variations, depending on whether the magnitude of the random displacement is large or small compared to the wavelength. We make use of this model because it is computationally efficient and, at the same time, it takes into account different channel effects, such as scattering, multipath propagation and Doppler shifting.

In this model, the attenuation of the channel is expressed as:

(1) $$A\left(d,f\right)=A_{0}\cdot d^{k}\cdot a{\left(f\right)}^{d},$$

where $A_{0}$ is a constant scaling factor, *f* is the frequency, *d* is the distance, *k* is the spreading factor and a(f) is the absorption coefficient, computed using Thorp’s empirical formula [20].

In an underwater channel, there will be multiple paths between the transmitter and the receiver—for instance, there will be reflections with the water surface. Moreover, due to waves and tides, the lengths of these paths will vary, which means that each path will have a different path delay and attenuation, as Equation (1) depends on the path length. In order to include these and other effects into account when modeling the underwater channel, the authors in [14] propose approximating the channel transfer function as follows:

(2) $$H\left(f,t\right)=H_{0}\left(f\right)\sum_{p}h_{p}\gamma_{p}\left(f,t\right)e^{-j2\pi f\delta_{p}},$$

where we have that:

- The paths are indexed by *p*, where $H_{0}$ denotes the reference path transfer function. The effect of each propagation path is considered as a low-pass filter, which takes into account both the attenuation expressed in Equation (1) and the reflections encountered along the propagation path.
- $h_{p}$ and $\delta_{p}$ are, respectively, the channel gain and delay for each path. They are obtained taking into account variations in the path length—for instance, due to tides.
- The coefficient $\gamma_{p}\left(f,t\right)$ includes other propagation effects, namely, scattering, correlation and Doppler shifting.

Note that Equation (2) depends both on the frequency and the time, hence the channel model used is not time-invariant. Even though it does not model some effects, such as surface curvature or the effect of breaking waves, it achieves a good compromise between being a realistic channel model and a computationally efficient one [21]. For these reasons, we use this channel model in our work.

### 2.2. Neural Networks

It has been known for many years that neural networks are universal approximators for functions, capable of approximating any measurable function to any degree of accuracy [22]. However, the lack of sufficient computational power for training these neural networks caused them not to be too successful. This radically changed as the computational power available to researchers grew: deep neural networks composed of many layers could be trained and many applications for DNNs appeared [23]. Today, DNNs are used successfully for many complex tasks, such as object classification in images [24], human-like performance in Atari games [25] or indoor localization [26], to mention a few. The increasingly high computational power available even makes it feasible to use DNN for WSN deployments [11].

**Figure 1:**
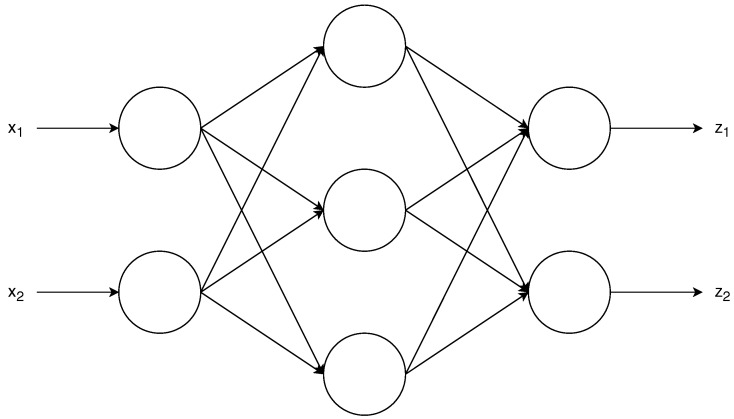
Example of feedforward neural network. Each circle represents a neuron, which combines in a nonlinear way its inputs following Equation (3). The inputs are $x_{1}$ and $x_{2}$, and the outputs are $z_{1}$ and $z_{2}$. There is a single hidden layer, which has three neurons. Note how each of the outputs *z* is a nonlinear combination of the inputs $x_{1}$ and $x_{2}$.

A feedforward neural network is a directed architecture as seen in Figure 1, in which each of the neurons outputs a nonlinear combination of its inputs as follows:

(3) $$z=F\left(\sum w\cdot x+b\right),$$

where *z* is the neuron output, *x* its input, *w* is a vector of weights, *b* is a scalar bias and *F* is the activation function, which usually is nonlinear. Note that each neuron receives as input a vector and outputs a single, deterministic value.

Training a neural network means obtaining the set of weights and biases that approximates a certain function. This is usually done from data: we provide the neural networks with a dataset of inputs and outputs, and the weights and biases are iteratively updated in order to minimize a loss between the neural network output and the desired output given by the dataset. This update is usually done by means of the backpropagation algorithm [27], which is an application of the chain rule to obtain the gradients and then apply a first order optimization method, such as Adam [28], in order to update the weights and biases of the neural network.

Feedforward neural networks can be modified to include feedback from the past, giving way to Recurrent Neural Networks (RNNs). RNNs are very suited for processing a stream of data, as they are able to remember past information. The memory is achieved by updating as new data arrives, a state, which is then concatenated to the neural network input. Hence, the output now is not only a function of the neural network input data, but also a function of the state, which contains information about the past.

One of the most popular architectures to implement an RNN is the Long-Short Term Memory (LSTM) architecture [29], which we use in our work. In an LSTM, there is a cell state $c_{t}$ that is updated using the data input $x_{t}$ and the output of the LSTM, denoted by $y_{t}$. The subscript *t* is used to denote the time index. An LSTM is formed by four different neural networks, with weights $w_{i}$ and biases $b_{i}$, i∈{1,2,3,4}—see Equation (3). These four neural networks are used to update $y_{t}$ and $c_{t}$ as follows:

- First, $c_{t}$ is updated with the following expression:
  (4) $$c_{t}=Sigm\left(w_{1}\cdot \left[x_{t},y_{t-1}\right]+b_{1}\right)\cdot c_{t-1}+Sigm\left(w_{2}\cdot \left[x_{t},y_{t-1}\right]+b_{2}\right)\cdot Tanh\left(w_{3}\cdot \left[x_{t},y_{t-1}\right]+b_{3}\right),$$
  where Sigm denotes the sigmoid function, Tanh the hyperbolic tangent function and [a,b] the concatenation of the vectors *a* and *b*. Note that the cell state $c_{t}$ is updated using the previous cell state $c_{t-1}$, the previous LSTM output $y_{t-1}$ and the current input $x_{t}$. The first term in Equation (4) is called the forget term: the sigmoid function outputs a nonlinear combination of the current input and previous output in the range [0,1]. By multiplying this term element-wise with the previous cell state, we are determining which elements from the previous cell state are forgotten.
  The second term in Equation (4) intuitively controls what new information we are adding to the cell state. Note that the hyperbolic tangent term could be considered the new information that the LSTM wants to add to the cell state, whereas the sigmoid term controls again how much of that information will be added to the cell state. Thus, the cell state update consists of two main terms: the first controls how much information from the previous state cell is remembered, and the second how much information from the current input and previous output we are adding to the state cell to remember in the next timesteps.
- Second, we obtain the output to the LSTM using the following expression:
  (5) $$y_{t}=Sigm\left(w_{4}\cdot \left[x_{t},y_{t-1}\right]+b_{4}\right)\cdot Tanh\left(c_{t}\right),$$
  where the input depends on the current input and cell state, and the previous output. Note that the cell state is updated using Equation (4) prior to obtaining the LSTM output using Equation (5). In addition, note that Equation (5) shows that the output is a filtered version of the current cell state.

**Figure 2:**
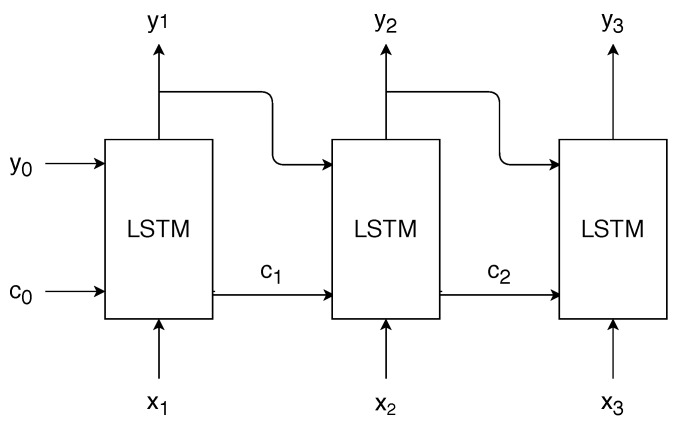
Illustration of the procedure of an LSTM for three timesteps. The output $y_{t}$ is updated in each timestep using Equation (5) and the cell state $c_{t}$ is updated using Equation (4). The LSTM block is composed of four neural networks, which are the same for all timesteps. Note that, in the first timestep, it is necessary to provide an initial $c_{0}$ and $y_{0}$ in order to obtain $c_{1}$ and $y_{1}$.

It is important to note that, in each timestep, the weights and biases of the four neural networks that compose the LSTM architecture are the same. The training of RNNs is different from feedforward neural networks, as now the time needs to be taken into account as well, and a modified algorithm known as backpropagation through time is used [30]. This algorithm computes the gradient for the weights and biases, not only taking into account the current timestep, but also the previous ones, unrolling the LSTM in a similar way as shown in Figure 2. Thus, sequences of input data are used for training an LSTM.

## 3. Problem Setup

In this work, we test a DNN architecture that can be used for localization in an underwater acoustic channel, for non-cooperative targets and without the need of using anchor nodes. We now proceed to detail the different elements that are part of our problem setup.

### 3.1. Target Signal Model

First, we consider that the target that we want to locate is an underwater vehicle, and model the signal that vehicle produces as the following sinusoid:

(6) $$x\left(t\right)=\cos\left(2\pi f_{m}t\right),$$

where x(t) is the acoustic signal generated by the vehicle and $f_{m}$ models the effect of the propeller blade. This simple signal model appears in underwater localization methods, such as [31], and in [32]; it is empirically shown that the propeller blades spectrum presents peaks around multiples of the blade rates. We do not study more complex signal models in this work, such as cavitation [33], in order to focus on the study of the influence of the channel characteristics on the localization accuracy.

### 3.2. Received Signal Model

We consider an underwater sensor network composed of *S* sensors, where s=1,2,…,S indexes the sensor. As indicated in Section 2.1, we choose to use the channel model from [14] because it is an accurate and computationally efficient acoustic channel model [21,34]. Note that the channel modeled in [14] is not time invariant, thus we make use of two different time-scales: τ represents a small time-scale, in the order of milliseconds, whereas *t* represents the large time-scale, on the order of seconds. Recall that, in a time variant convolution, the channel impulse response depends on *t*, and that the signal $y_{s}\left(t\right)$ in each underwater acoustic sensor *s* can be obtained as the convolution in τ of the channel impulse response in the corresponding *t* for the sensor *s*, $h_{s}\left(t,\tau \right)$, plus a term of additive white Gaussian noise at the receiver, n(t). Mathematically:

(7) $$y_{s}\left(t\right)=x\left(t\right)*h_{s}\left(t,\tau \right)+n\left(t\right),$$

where ∗ denotes the convolution operator. We remark that x(t) is defined in Equation (6).

For obtaining $h_{s}\left(t,\tau \right)$, we use the model from [14]. For each different *t*, we generate a new channel realization: note that this means that the statistical effects of the channel will vary with *t*, as, for each *t*, we will have a different channel realization. The model in [14] is defined using a large set of parameters that are found in Table 1. Note that we choose many parameters to be dependent on a θ value, where θ∈[0,1] controls the variability of the channel, i.e., the channel presents the highest variability when θ=1 and the opposite when θ=0. When θ=1, the parameters’ values are the default ones in [35].

### 3.3. Covariance Matrix Feature Extraction

In previous works [12,13], the authors have made use of a DNN to locate targets using values from the covariance matrix in order to feed a deep neural network. In order to obtain the covariance matrix *C*, we first use the received signal $y_{s}\left(t\right)$ sampled during a certain time *T* to obtain the FFT vector for each sensor, $Y_{s}$. Note that *T* is the time interval considered for all signal processing tasks in this work. We then obtain *Y*, which is an *S*-component vector, in which each entry corresponds to the normalized FFT value in the frequency bin with maximum energy for each sensor *s*. In other words:

(8) $$Y\left(s\right)=Y_{s}\left(\arg\max|Y_{s}{|}^{2}\right).$$

The covariance matrix is obtained using the following expression:

(9) $$C=Y\cdot Y^{H},$$

where *H* is the conjugate transpose operator. Note that $Y_{s}$ and *Y* are complex vectors, and *C* is a complex Hermitian matrix—that is, a square matrix that is equal to its own conjugate transpose.

The covariance feature vector is obtained from the *C* matrix as follows. First, we take advantage of the fact that *C* is Hermitian in order to reduce the dimensionality of the feature vector: we only need to take the upper (or the lower) triangle values of the matrix. In our case, we take the upper triangle values and concatenate them, hence we have a vector *c* with (S−1)S/2 components, instead of $S^{2}$ components of the original *C* matrix.

**Figure 3:**
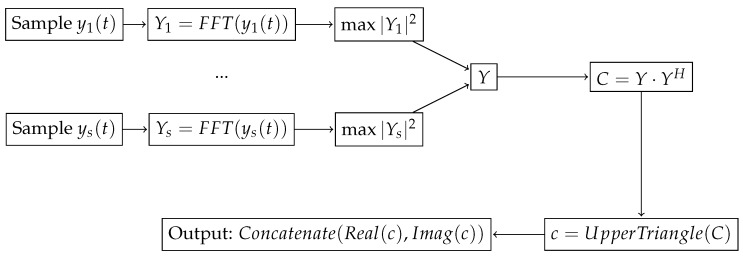
Block diagram illustrating the steps needed to obtain the covariance feature vector.

However, each component of the vector *c* is complex, and the neural network needs real numbers as input. Thus, we concatenate the real and imaginary parts of *c* in order to obtain the covariance feature vector, which has a dimensionality of S(S+1) components. Note that, if we did not take into account the fact that *C* is a Hermitian matrix, the feature vector would have $2S^{2}$ components. The whole procedure can be observed in Figure 3.

Finally, note that *C* includes information about the received signal power and phase, and hence the feature vector includes both of them.

### 3.4. Power Feature Extraction

In this work, we make use of the power as another possible localization feature. In this case, we consider that the input to our localization DNN is an *S*-component vector, in which the component for each sensor *s* is $E_{s,t}$, the received signal energy during *T*, that is:

(10) $$E_{s,t}=\int_{t}^{t+T}{\left|y_{s}\left(u\right)\right|}^{2}du.$$

In other words, we choose as an input feature for our localization DNN the signal energy that each sensor *s* is measuring. Note that, in this case, the feature vector has a size *S*, which is significantly lower than in the covariance features. In addition, note that the covariance includes phase information, while the power approach does not. In addition, note that the main diagonal of the matrix *C* includes energy information as well.

### 3.5. Localization Neural Network

**Figure 4:**
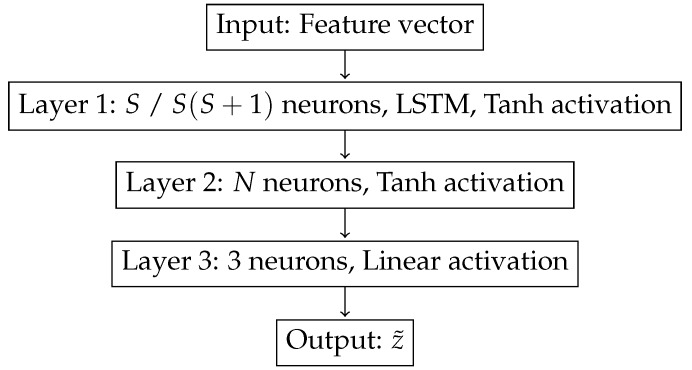
The localization DNN architecture used, where Tanh stands for hyperbolic tangent function. The number of neurons of the first layer depends on the dimensionality of the feature vector used: *S* neurons if power features are used, and S(S+1) if covariance features are used.

In order to locate, we use a neural network with one single hidden layer that can be observed in Figure 4. The first layer has one neuron for each component of the feature vector, that is, *S* neurons if power features are used, and S(S+1) if covariance features are used. This first layer is an LSTM cell, which, as we indicated previously, is specially suited to analyze a sequence of data. The second layer is a feedforward layer with *N* neurons: we will test different values of *N* in order to study how the number of neurons of the hidden layer affects the localization precision. We denote the actual position of the vehicle by *z*, which in this work is a three-dimensional, vector with the Cartesian coordinates of the vehicle. Hence, the last layer of the network has three components and it is denoted by $\tilde{z}$, an estimation of the localization of the vehicle target. Observe that our method is valid to estimate the localization of surface and underwater targets. In addition, note that the activation functions chosen for the first two layers are the hyperbolic tangent function, but the final layer has a linear activation. This is due to the fact that the hyperbolic tangent function gives values in the range [−1,1], while the position vector *z* takes values outside that range.

**Figure 5:**
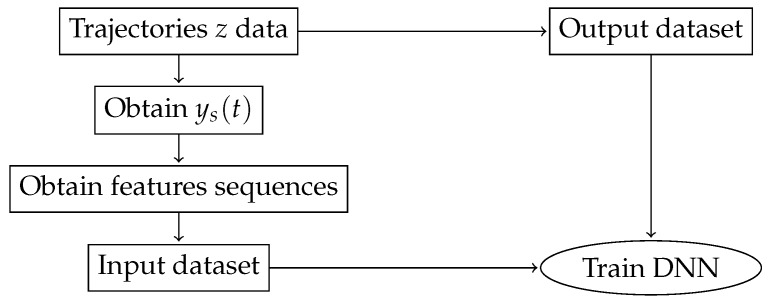
The localization procedure overview. From a set of trajectories, we obtain the signal received in each sensor *s*, $y_{s}\left(t\right)$, which is then used to extract power or covariance features, and these features are grouped in sequences to form the input data to our localization DNN. The output data are the trajectory Cartesian coordinates *z*. By training the DNN in this way, we minimize the error between the predicted localization by the localization DNN, $\tilde{z}$, and the actual localization *z*.

In order to train the neural network, we first generate a set of trajectories *z*. For each point in these trajectories and for each sensor *s*, we use our channel model to generate the channel impulse response H(f,t), using Equation (2), and this impulse response is used to obtain $y_{s}\left(t\right)$, the signal received at each sensor using Equation (7). Then, we extract the features from the received signal, which depend on whether we are using the covariance of the received signal—Equation (9)—or the power received—Equation (10). Since our localization DNN has an LSTM, the input dataset is formed by sequences of consecutive features, in order to allow the LSTM train its memory capabilities. The output dataset is composed by the *z* localization information, that is, the actual localization of the target. Thus, we have a sequence of features at the input, and the output is an estimation of the position of the target. We train the DNN by minimizing the mean squared error between *z*, the actual target localization, and $\tilde{z}$, the localization estimation given by the DNN. By training in this way, we minimize the error between the predicted localization, $\tilde{z}$, and the actual localization *z*. A block diagram of this process can be seen in Figure 5.

Finally, we comment on another advantage of using a recurrent neural network to locate as we do. It is known that such neural networks are able to track moving targets in an unsupervised way [15]. Hence, the inclusion of an LSTM layer in our localization DNN means that the predicted target localization, $\tilde{z}$, will be a filtered version that takes into account past information, in a similar way to other well known tracking procedures, such as Kalman filters. Thus, using LSTM provides an additional advantage, which is that our localization DNN is also tracking using past information, as [15] shows.

## 4. Results

Now, we proceed to test the performance of our proposed DNN. We first describe the test conditions, and then present and analyze the results obtained, using covariance features as a baseline to compare the performance of power features.

### 4.1. Simulations Setup

We use the setup described in Section 3 in order to train the proposed localization DNN. First, we work with discrete time signals, using as sample frequency $f_{s}$ = 10 kHz. We generate x[n] following Equation (6) with $f_{m}$ = 250 Hz using 1024 samples.

**Figure 6:**
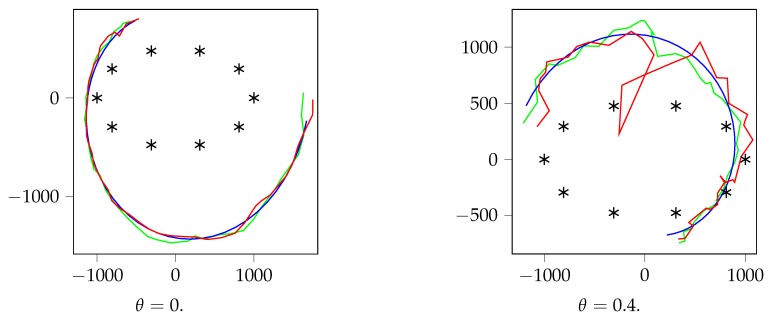
Example trajectories, for SNR=0 dB and N=64. The black points represent the sensor positions. Blue is the actual value of *z*, and, in red, we observe the localization DNN prediction, $\tilde{z}$, using power features, and, in green, using covariance features. The axis are in *m*.

Next, we place S=10 sensors in an ellipse whose semi-major axis measures 1000 m and the semi-minor axis measures 500 m: we consider these sensors to be static. In addition, we generate 100 trajectories, with 50 points in each of them: each trajectory follows a two-dimensional spiral with randomly picked phase, radius and angular velocity in the water surface. We consider that the time spacing between the trajectory points is 6 s. With regard to the depth of sensors and target, we consider that the sensors lie at 1 m height of the sea bottom, whereas the target moves at a height of 50 m, which corresponds to the sea surface. The Cartesian coordinates of the target is the vector z=(x,y,50), where:

(11) $$\begin{array}{cc} x[n] & =x_{0}+n\cdot R\cdot \cos\left(\omega \frac{n}{50}+\phi \right),\\ y[n] & =y_{0}+n\cdot R\cdot \sin\left(\omega \frac{n}{50}+\phi \right), \end{array}$$

where *x* and *y* are in *m*, $x_{0}$ and $y_{0}$ are uniformly chosen in the range [0, 1000] m, *R* is uniformly chosen in the range [100, 1100] m and ω and ϕ are uniformly chosen in the range [0,2π]. We use these trajectories for they describe realistic target trajectories. See Figure 6 to see some examples of the trajectories obtained. Note that this model allows a very convenient parameterization, which faces our localization DNN to a complex set of curved trajectories around the sensors.

After that, we obtain the channel impulse response from each point of each trajectory to each sensor *s* using the procedure described in [14] with the parameters from Table 1 for 11 equispaced θ values in θ∈[0,1]. Since the channel is time variant, we obtain a different channel impulse response for each trajectory point. The signal x[n] is filtered using the corresponding channel impulse response in order to obtain $y_{s}\left[n\right]$ as in Equation (7). We also add to $y_{s}\left[n\right]$ an additive white Gaussian noise, whose energy depends on the Signal-to-Noise Ratio (SNR) that we fix beforehand using three different values: SNR={0,10,20} dB. For each point *z* in each trajectory, we generate 100 different $y_{s}\left[n\right]$ values for two reasons: to have a larger dataset, which helps with training the DNN, and to help the DNN deal with the effect of the Gaussian noise that we add at the receiver.

In order to obtain the features, we use 40 ms of the received signal. The covariance features are obtained using Equation (9), and the power features are obtained using Equation (10) in logarithmic units. We remark that we use only 40 ms of the signal $y_{s}\left[n\right]$ to obtain the energy. Since our localization approach relies on the signal energy, it is important to obtain the energy from a number of samples that is high enough as to guarantee an accurate estimate of the signal energy, but, at the same time, it should not be too large in order to ease the computational load and cause the signal to be locally time invariant. For our case, we found that 40 ms were enough for our purposes.

The features obtained are the input to the localization DNN. We normalize the input data, so that it approximately has a zero mean and a unit variance, as normalization has been observed to improve significantly the performance of DNNs [36].

We are now ready to start training the localization DNN. We divide each trajectory into sequences of 10 consecutive positions: these sequences will form our input dataset. In addition, 90% of the dataset is used to train the DNN, and the other 10% is used for validation, i.e., samples that the DNN has never seen. We train the localization DNN during 1000 epochs, using the batch of data generated, trying to minimize the mean squared error between *z* and the prediction of *z*, $\tilde{z}$ that the localization DNN provides. As optimization algorithm, we use Adam [28] with a learning rate of 0.001. This training procedure is repeated using a different number of neurons in the hidden layer, N∈{8,16,32,64,128}.

### 4.2. Results Obtained

**Figure 7:**
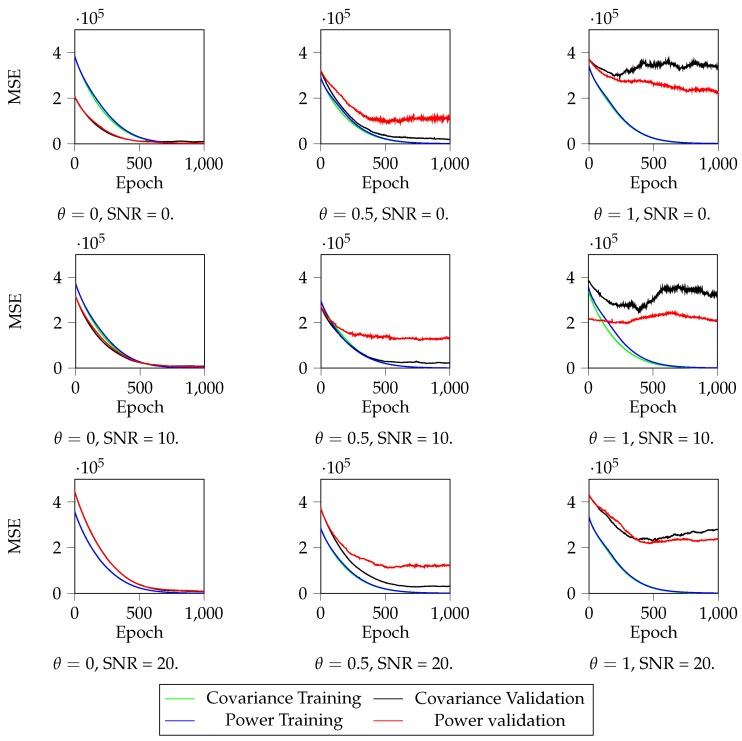
Mean Squared Error (MSE) during training, when N=64. We can observe that, for low θ and high SNR, the localization DNN converges to a low localization error. However, as θ increases, i.e., the channel becomes more variable, and SNR diminishes, i.e., the received noise signal increases, the localization precision significantly worsens. In addition, as θ increases and SNR diminishes, there appears overfitting: the validation values remain constant while the training values decrease, which means that there is a certain localization precision bound that our localization DNN is not able to improve.

**Figure 8:**
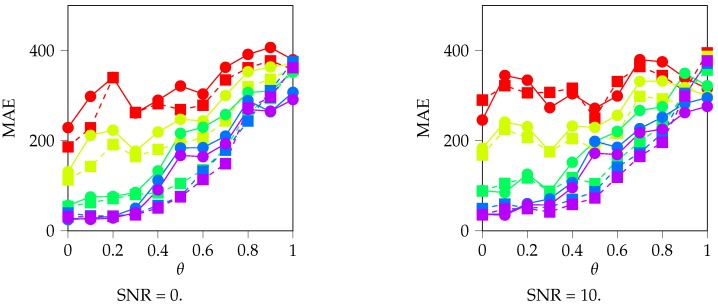

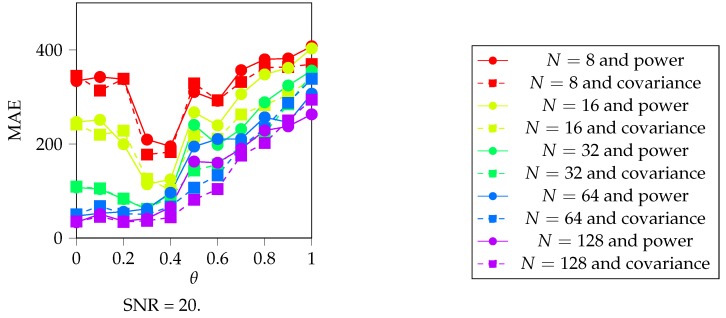
Validation Mean Absolute Error (MAE), in *m*, for different θ, SNR and *N*. We use the MAE because its meaning is more intuitive than MSE as the mean error in the distance between *z* and $\tilde{z}$. Note that, as θ increases, the MAE increases as well, specially from θ=0.4 onwards. We can also observe that, for N={8,16}, the localization DNN is not learning to locate accurately, which does for higher *N*, that is, for more neurons in the hidden layer. However, note that the increase in accuracy between N=64 and N=128 is minimal. Finally, observe that the results are similar for all SNR values and that, for low θ, using power or covariance features does not matter, but it does for higher θ values.

We analyze the results in Figure 6, Figure 7 and Figure 8. First, in Figure 7, we can observe the training and validation losses for the case that N=64. The results obtained show that the localization DNN learns very well for θ=0, but overfits a bit when θ=0.5 and a lot when θ=1, for all SNR values. Thus, we can conclude that θ, the parameter that controls the channel variability, is the main responsible for the localization accuracy. In the best case, the localization DNN learns in an accurate way, but, as the variability increases, the learning DNN reaches a precision bound and then it starts overfitting. Hence, for high θ values, the training should stop early in order to avoid the DNN to overfit, that is, stop training when the validation error stops decreasing. In addition, note that the effect of the SNR on the localization precision is negligible compared to the θ effect: higher SNR produces a better localization, but clearly θ dominates the localization precision.

In Figure 8, we can observe the validation Mean Absolute Error (MAE) compared for all of our experiments. We choose to represent the MAE because it gives a more intuitive insight into the meaning of the error value: the MAE represents the mean distance in each coordinate between *z* and $\tilde{z}$, that is, between the actual and the estimated localization of the target. Figure 8 allows us to study the effect of each of the parameters under study and their effect on the localization precision:

- Regarding *N*, the number of neurons of the hidden layer, we note that having more neurons does benefit our localization precision, as it decreases the MAE. Actually, for N=8 and N=16, the localization DNN does not learn to locate, but it does for N≥32. We remark that, while adding more neurons does increase the localization precision, the marginal increase in the MAE is low when we pass from 64 to 128 neurons. In addition, note that there is a lower bound in the localization precision for all values, which is around 50 m in our setup, due to the uncertainties in the localization that are derived from the channel propagation effects.
- Regarding θ, we reach the same conclusion as in Figure 7: as θ increases, the precision localization decreases, as the MAE increases. Note that this affects N≥32, all SNRs and both power and covariance features. This is no surprise at all: as we showed in the Introduction, the main obstacle to using power measures for locating purposes is precisely the complexities of the underwater channel.
- Regarding the SNR, we note that it does not play a significant role in determining the localization precision in our setup, as the plots are similar for all SNR cases.
- Regarding the features used, note that, for low θ values, covariance and power features reach a similar localization precision, which is to be noted as the power feature is a single scalar per sensor, while covariance is a vector. However, as θ increases, power features’ localization precision has a faster increase in the MAE, which means that covariance locates better, although the localization results are not good for any of these features. These results are to be expected: covariance features include more information than power features, and hence their performance should not be worse than power. It is remarkable, however, that power features include enough information for locating accurately when the channel variability is low.

Finally, Figure 6 shows, for N=64 and θ=0 and 0.4, two examples of validation trajectories and the prediction given by the localization DNN. It is possible to observe that, if the variability of the channel is not too large, i.e., θ is low, the localization DNN provides a good localization mechanism. Note that θ will depend on the concrete setting where we intend to deploy our locating sensor network, and thus locating using power or covariance and a DNN could be a good option. As we have indicated, using a localization DNN provides several other advantages.

One of them has to do with whether the sensors need to know their positions. It is possible to differentiate between two cases. If the sensors are deployed first, and then used to collect real data from sample trajectories done with a moving target, the sensors need not know their exact positions, as the localization DNN will implicitly learn everything it needs for localization during training. However, a different possible approach would be to train the localization DNN using simulations, as we have done, and then deploy the sensor network. In this case, the sensors need to be positioned in the same geometry that was used for training, as the localization DNN has implicitly learned to locate using that concrete geometry. Note that both methods could be also combined in order to pre-train the localization DNN weights and then fine-tune them with real data. Thus, depending on which training procedure we choose, our method may need the sensors to know their position.

In addition, our method does not need the cooperation of the target: there is no messaging scheme required to locate between the target and the sensor network, as it is the case with Time of Arrival based methods. However, communication is needed among the sensors in order to locate. Note that the data that are transmitted among sensors depend on the features that are used: if we are using power features, the sensors only transmit a scalar. However, if the localization DNN is using covariance features, then each sensor has to transmit the signal that each of them has received in order to extract the covariance features. Hence, the communication requirements in the case of power features are significantly smaller than in the case of using covariance features.

The final advantage is that our localization DNN does not need to explicitly model the underwater channel either, as the DNN infers the relation between the energy and the positions during training. However, if we want to train the localization DNN using simulations, we need to have access to a channel simulator in order to obtain samples for training.

We also note that our approach presents some disadvantages that need to be taken into account. The main one is that a training process is required in order to make the system operative, which means deploying the sensors and recording data from some example trajectories to do the training or to have access to a channel simulator. Our method also requires that sensors are fixed; otherwise, the training phase should be repeated and it requires communication among the sensors. Note that, if the training is performed with real data, with the WSN deployed, the training would also bring the advantage of adaptability because the localization DNN will learn to locate implicitly using the concrete geometry of the zone. This is an advantage that anchorless methods such as [9] also have, with the important difference that we do not need to map the sea region since the localization DNN will infer that by itself during the training phase, considerably reducing the computational load.

## 5. Conclusions

In this work, we have studied the performance of a DNN architecture for locating in an underwater environment using acoustic signals emitted by a moving target. We have proposed a concrete architecture that makes use of memory to locate the target, and we have used an underwater channel model which represents the most dominant propagation effects, such as multipath propagation, scattering or Doppler shifting. We compare the localization precision for different conditions of SNR at the receiver, channel variability, number of neurons in the localization DNN and for two different kinds of features, using the power and the covariance of the acoustic signals. We show that, if the channel variability is low, it is possible to use a localization DNN with power or covariance features to successfully locate a target. We remark that, under such conditions, power yields a similar performance to covariance, being simpler to obtain and with a lower dimensionality, which significantly helps the localization procedure as there will be fewer communications among sensors. Hence, our work shows power can be used successfully as a localization mechanism in the underwater channel under certain conditions.

This work could be extended in several ways. One of them could be to go deeper into the localization DNN architecture and study if some parameters that we have kept fixed affect the localization performance. For instance, it would be interesting to check whether the number of hidden layers or the sequence length used to feed the LSTM affect the localization precision. Another possible extension consists of modifying the target signal, using more complex models, and studying whether other feature extraction methods, such as cyclostationary information [37], can be used for localization.

## Figures and Tables

**Table 1:** Channel parameters used, following the channel model from [14]. Note that there are values that depend on the θ parameter chosen: higher θ values provide a channel with more variability. Thus, we use θ to model acoustic channels with different variability while using a single parameter for simplicity.

Parameter	Value
Spreading factor *k*	1.7
Speed of sound in water (m/s)	1500
Speed of sound in bottom (m/s)	1200
Minimum relative path strength	50
Frequency band (kHz)	[10,20]
Frequency resolution (Hz)	25
Coherence time small scale $T_{SS}$ (s)	6
Variance of small scale surface variations $\sigma_{s}^{2}$ (m${}^{2}$)	1.125θ
Variance of small scale bottom variations $\sigma_{b}^{2}$ (m${}^{2}$)	0.5625θ
3-dB width of psd of intrapath, $B_{\delta p}$ (Hz)	0.0005
Number of intrapaths	20θ
Mean of intrapaths amplitudes	0.025θ
Variance of intrapaths amplitudes	0.000001θ
Range of surface height (m)	[−θ,θ]
Range of target height (m)	[−θ,θ]
Range of sensor height (m)	[−θ,θ]
Range of channel distance height (m)	[−10θ,10θ]
Large scale standard deviation of surface height	θ
Large scale standard deviation of target height	θ
Large scale standard deviation of sensor height	θ
Large scale standard deviation of channel distance	θ
Large scale auto regressive process parameter	0.9

## Abbreviations

The following abbreviations are used in this manuscript:

WSN	Wireless Sensor Network
AUWSN	Acoustic Underwater Wireless Sensor Network
DNN	Deep Neural Network
SNR	Signal-to-Noise Ratio
RNN	Recurrent Neural Network
LSTM	Long-Short Term Memory
MSE	Mean Squared Error
MAE	Mean Absolute Error
